# Reliability and interrelations of seven proxy measures of cochlear synaptopathy

**DOI:** 10.1016/j.heares.2019.01.018

**Published:** 2019-04

**Authors:** Hannah Guest, Kevin J. Munro, Garreth Prendergast, Christopher J. Plack

**Affiliations:** a Manchester Centre for Audiology and Deafness, University of Manchester, Manchester Academic Health Science Centre, UK; b Manchester University NHS Foundation Trust, UK; c Department of Psychology, Lancaster University, UK

**Keywords:** Cochlear synaptopathy, Hidden hearing loss, Auditory nerve, Auditory brainstem response, Envelope-following response, Middle-ear-muscle reflex, ABR, auditory brainstem response, AN, auditory nerve, AP, action potential, CF, characteristic frequency, EFR, envelope-following response, ICC, intraclass correlation coefficient, MEMR, middle-ear-muscle reflex, RMS, root mean square, SD, standard deviation, SNR, signal-to-noise ratio, SP, summating potential, SR, spontaneous rate

## Abstract

Investigations of cochlear synaptopathy in living humans rely on proxy measures of auditory nerve function. Numerous procedures have been developed, typically based on the auditory brainstem response (ABR), envelope-following response (EFR), or middle-ear-muscle reflex (MEMR). Validation is challenging, due to the absence of a gold-standard measure in humans. Some metrics correlate with synaptic survival in animal models, but translation between species is not straightforward; measurements in humans are likely to reflect greater error and greater variability from non-synaptopathic sources. The present study assessed the reliability of seven measures, as well as testing for correlations between them. Thirty-one young women with normal audiograms underwent repeated measurements of ABR wave I amplitude, ABR wave I growth, ABR wave V latency shift in noise, EFR amplitude, EFR growth with stimulus modulation depth, MEMR threshold, and an MEMR across-frequency difference measure. Intraclass correlation coefficients for ABR wave I amplitude, EFR amplitude, and MEMR threshold ranged from 0.85 to 0.93, suggesting that such tests can yield highly reliable results, given careful measurement techniques. The ABR and EFR difference measures exhibited only poor-to-moderate reliability. No significant correlations, nor any consistent trends, were observed between the various measures, providing no indication that these metrics reflect the same underlying physiological processes. Findings suggest that many proxy measures of cochlear synaptopathy should be regarded with caution, at least when employed in young adults with normal audiograms.

## Highlights

• Given careful measurement techniques, ABR and EFR amplitudes can be highly reliable.
• The same is true of MEMR thresholds and MEMR across-frequency threshold difference.
• Differential ABR and EFR measures exhibit only poor-to-moderate reliability.
• Correlations between measures are not evident in young people with normal audiograms.
• Proxy measures of synaptopathy in this population should be regarded with caution.

## 1 Introduction

Seminal work in a mouse model demonstrated that noise exposure can destroy synapses between cochlear inner hair cells and auditory nerve (AN) fibers, without widespread hair-cell loss (Kujawa and Liberman, 2009). Direct evidence of cochlear synaptopathy has since been observed in rats, guinea pigs, gerbils, and macaques (Hickox et al., 2017; Valero et al., 2017). In affected animals, cochlear thresholds are left largely intact, yet auditory brainstem response (ABR) amplitudes are reduced at moderate-to-high stimulus levels (Hickox et al., 2017). Synaptopathy may preferentially affect the subset of AN fibers with low-to-medium spontaneous firing rates (low-SR fibers; Furman et al., 2013), which have high response thresholds (Liberman, 1978). It is thought that the pathophysiology might have significant perceptual consequences, including deficits of speech perception in noise and tinnitus (Plack et al., 2014).

Naturally, researchers worldwide have hastened to investigate whether cochlear synaptopathy manifests in humans. The vast majority of studies have not employed histological techniques, relying instead on non-invasive proxy measures of cochlear synaptopathy. A wide variety of such measures are reported in the literature, generally based on the ABR, envelope-following response (EFR), or acoustic middle-ear-muscle reflex (MEMR).

Most widely used is the amplitude of ABR wave I, which reflects the summed activity of AN fibers (Melcher and Kiang, 1996). When measured in response to high-level stimuli, wave I is assumed to include substantial contributions from high-threshold fibers. Accordingly, in animal models of synaptopathy, wave I amplitude is reduced at medium-to-high stimulus levels (Kujawa and Liberman, 2009). However, sensitivity of the measure to low-SR damage has been questioned, due to the delayed onset responses of this fiber type (Bourien et al., 2014). Moreover, ABR amplitudes in humans exhibit greater between-subject variability than in rodents, attributable to many factors unrelated to synaptopathy, such as head size, sex, cochlear dispersion, physiological noise, and pre-neural dysfunction (Plack et al., 2016). Within-subject difference measures may be of value in managing this variability.

One candidate measure is the ratio of wave I amplitude to wave V amplitude (Schaette and McAlpine, 2011). Sensitivity of the measure to synaptopathy rests on the assumptions that (i) the amplitudes of both waves are equally affected by non-synaptopathic sources of variability, and (ii) the amplitude of wave V is not reduced by synaptopathy, due to enhanced neural gain in the auditory brainstem. The first assumption, in particular, may not hold, since the two waves differ in terms of the cochlear regions represented in the response. Experiments using high-pass masking have shown that wave I is dominated by contributions from high characteristic frequencies (CFs), including those >8 kHz, while wave V is dominated by CFs <2 kHz (Don and Eggermont, 1978; Hardy et al., 2017). Consequently, the wave I/V amplitude ratio may be highly sensitive to high-frequency audiometric deficits. Since the clinically normal audiometric range spans 30 dB, “normalization” of wave I by wave V may not be meaningful, even in humans with normal audiograms.

An alternative difference measure, obtained using intra-canal electrodes, is the ratio of summating-potential amplitude to action-potential amplitude (SP/AP ratio; Liberman et al., 2016). Since the SP is a pre-synaptic potential, Liberman and colleagues reasoned that it should provide a means of normalizing the AP (equivalent to ABR wave I). However, this rationale requires that the SP and AP be similarly affected by differences in pre-neural function, which may not be so. Generation of the SP is complex, featuring contributions from inner and outer hair cells that vary across frequency and level (Durrant et al., 1998). It is important to understand how these potential sources of variability affect the resulting difference measure, especially if it is to be applied in humans with known audiometric deficits, as in Liberman et al. (2016). Use of the measure is further complicated by the extreme stimulus levels required to reliability elicit the SP (130 dB peSPL in the work of Liberman and colleagues).

A more straightforward measure is growth of ABR wave I amplitude with stimulus level. In a number of animal models of synaptopathy, ABR wave I growth functions have been shown to exhibit abnormally shallow slopes, with the greatest decrements in amplitude observed at the highest stimulus levels (e.g. Kujawa and Liberman, 2009). However, a potential practical issue is that measurement requires that ABR wave I be consistently evident at a range of stimulus levels, without exceeding comfortable loudness levels. Some researchers have questioned whether this is feasible, even in young humans with normal hearing (Mehraei et al., 2016).

In light of concerns about wave I measurement, Mehraei and colleagues devised a latency-based ABR measure: the shift in wave V latency with increasing background noise level. Sensitivity to synaptopathy rests on the assumption that the (low-SR) fibers most affected by synaptopathy have both longer response latencies and greater resistance to noise masking than the remaining fibers. Hence, in healthy ears, increasing noise levels should lead to rapidly increasing dominance of the ABR by low-SR fibers, causing ABR latency to increase markedly with noise level. In synaptopathic ears, which are assumed to have fewer low-SR fibers, the increase in latency should be less pronounced. Consistent with this reasoning, Mehraei et al. (2016) demonstrated smaller latency shifts in mice with noise-induced synaptopathy than in controls. A parallel experiment in humans showed that the measure was correlated (*p* = 0.036, uncorrected) with ABR wave I growth. However, in light of the small sample size (n = 10), replication is required.

Less commonly used than the ABR is the envelope-following response (EFR), a sustained neural response to the envelope of an amplitude-modulated (AM) stimulus. Since synchronization to AM tones is particularly strong in low-SR fibers, it has been suggested that the EFR might feature greater contributions from low-SR fibers than the transient-evoked ABR (Shaheen et al., 2015). Accordingly, some animal models have demonstrated good sensitivity of EFR measures to synaptopathy, though only at high stimulus modulation rates, around 1 kHz (Shaheen et al., 2015; Parthasarathy and Kujawa, 2018).

Human synaptopathy research has also employed a variety of EFR difference measures, such as change in EFR amplitude with increasing level of background noise or with modulation depth (Bharadwaj et al., 2015). As with the ABR difference measures, these metrics aim to reduce variability from non-synaptopathic sources, based on assumptions about the relative contributions of high- and low-SR fibers in the various stimulus conditions. However, to our knowledge, such measures lack validation in animal models. Indeed, Parthasarathy et al. (2017) found that EFRs at a range of modulation depths were equally impacted by synaptopathy.

A more recent addition to the battery of potential measures of synaptopathy is the acoustic MEMR: the involuntary contraction of the stapedius muscle in response to high-level sound stimuli. The afferent portion of the reflex arc may be driven by medium- and low-SR fibers (Valero et al., 2018). Accordingly, Valero and colleagues showed that cochlear synaptopathy in mice was more readily detected by MEMR measures than by ABR wave I amplitude. Sensitivity was maximized by use of threshold (rather than amplitude) measures, and by use of a high-frequency narrowband (rather than broadband) elicitor.

In summary, numerous procedures have been suggested and/or employed as proxy measures of cochlear synaptopathy in humans. The notion that all measures reflect the same underlying physiological processes is untested. Moreover, the use of multiple measures and conditions, even within a single study, inflates the potential for false discovery, if appropriate correction is not undertaken. These issues complicate interpretation of existing synaptopathy data in humans. Clarification of the situation is made challenging by the absence of a gold-standard, direct measure of synaptopathy in living humans. One important course of action should be thorough validation of promising measures in appropriate animal models, ideally in primates. Another is to investigate the reliability of measures in humans, to determine their capacity to discriminate among members of the target population despite measurement error. A third is to observe the extent to which the measures correlate, since correlations should be observed among sensitive measures of synaptopathy. The present study aims primarily to assess the reliability of seven proxy measures of synaptopathy (see Table 1), with a secondary aim of describing correlations between measures.

**Table 1:** Proxy measures of cochlear synaptopathy.

Measure
ABR wave I amplitude
Growth of ABR wave I amplitude with stimulus level
ABR wave V latency shift in noise
EFR amplitude
Growth of EFR amplitude with stimulus modulation depth
Acoustic MEMR threshold
MEMR across-frequency threshold difference

## 2 Material and methods

### 2.1 Participants

Target sample size (following attrition) was set at 30, sufficient to estimate the intra-class correlation coefficient (ICC) for ABR wave I amplitude with a 95% confidence interval (CI) of ∼0.2. Participants were of a single sex, due to known sex differences in electrophysiological response amplitudes, which might otherwise confound correlations between measures (Plack et al., 2016). Forty-one women aged 18 to 30 were recruited via advertising on the University of Manchester website. They reported no history of middle-ear surgery, hearing loss, neurological disorder, ototoxic exposure, or head trauma. Ten were excluded due to presence of wax in the ear canal (n = 7), abnormal audiogram (n = 1), Eustachian tube dysfunction (n = 1), or self-reported sound intolerance (n = 1). The remaining 31 exhibited normal otoscopic findings, normal pure tone audiometric thresholds (≤20 dB HL at 0.25–8 kHz), and normal tympanometric results (compliance 0.3–1.6 cm^3^, pressure −50 to +50 daPa). Their mean age was 24.4 years (standard deviation [SD] = 3.7 years). Of these 31, one failed to complete the second test session, due to the onset of Eustachian tube dysfunction. Hence, the sample size for the reliability analyses was 30, whereas all 31 were included when testing for correlations between measures.

### 2.2 Test sessions

Each participant attended two sessions, separated by at least 24 h and fewer than 30 days. Table 2 details session content. For 30 participants, the test ear for ABR and MEMR measurements was the left ear; for one participant, the right ear was selected, due to personal preference. All procedures were approved by the Human Communication, Development and Hearing Ethics Panel at the University of Manchester.

**Table 2:** Session content.

Session 1	Session 2
Screening questionnaire (5 min)	
Otoscopy (2 min)	Otoscopy (2 min)
Audiometry (10 min)	
Tympanometry (2 min)	Tympanometry (2 min)
DPOAEs (5 min)	
MEMR thresholds (10 min)	MEMR thresholds (10 min)
Preparation for electrophysiology (25 min)	Preparation for electrophysiology (10 min)
EFR at two modulation depths (30 min)	EFR at two modulation depths (30 min)
ABR at three noise levels, for latency-shift measure (25 min)	ABR at three noise levels, for latency-shift measure (25 min)
ABR at three click levels, for growth measure (35 min)	ABR at three click levels, for growth measure (35 min)

### 2.3 Pure-tone audiometry

Participants were seated in double-walled sound-attenuating booth. Pure-tone air-conduction thresholds were obtained from both ears in accordance with the recommended procedures of the British Society of Audiology (2011), using a Kamplex KC50 audiometer. Stimuli at 0.25, 0.5, 1, 2, 4, 8 kHz were delivered via TDH-39 supra-aural headphones; stimuli at 11.2, 14, and 16 kHz were delivered via HDA300 circum-aural headphones.

### 2.4 Distortion-product otoacoustic emissions

Distortion-product otoacoustic emission (DPOAE) testing was implemented using an Otodynamics ILO292 measurement system. The frequency ratio of the primary tones, ƒ2/ƒ1, was 1.22. The levels of ƒ1 and ƒ2 were 65 and 55 dB SPL, respectively. The geometric mean of the pair was swept from 8000 to 500 Hz, with measurements conducted at two points per octave. Data collection terminated after three such sweeps.

### 2.5 Electrophysiological setup

Participants reclined with eyes closed in a double-walled, sound-attenuating booth. Auditory stimuli were presented via electromagnetically shielded EARtone ER1 insert earphones driven by an Avid FastTrack C400 audio interface and Canford Mk.2 headphone amplifier. A BioSemi ActiveTwo measurement system recorded from active electrodes at Cz, C7, and the mastoid of the stimulated ear. Common Mode Sense and Driven Right Leg electrodes were attached at mid-forehead and electrode offsets remained within ±40 mV throughout all recordings. Data streams from all three electrodes were saved for offline analysis, along with stimulus-timing information received from the external audio interface via a custom-made trigger box.

Previous researchers have reported difficulty in obtaining clear responses in some of our electrophysiological measures (e.g. Mehraei et al., 2016). The present study adopted a three-fold approach to this issue. Firstly, extensive piloting and optimization of all measures was used to determine stimulus parameters that would produce clear responses in most young people with normal hearing. Achieving this result without using very high sound levels was made a priority, not least because comfortable stimuli may be expected to relax participants and reduce physiological noise. Secondly, the recording environment was carefully prepared to maximize participant comfort: lights were dimmed; air temperature was maintained at 25 °C; a chair was selected which reclined to horizontal and offered excellent neck support; a choice of blankets was offered, to ensure a comfortable body temperature for each individual. Finally, extensive pre-recording counselling was provided (lasting ∼15 min), with the aim of allaying any potential concerns and uncertainties. Counselling addressed the following issues: (i) purpose and basic setup of the recording; (ii) skin preparation, electrode placement, electrode removal, and clean-up; (iii) ear tip insertion and associated sensations; (iv) duration of each stimulus; (v) sound quality of each stimulus, described candidly; (vi) the need for a relaxed state during the majority of the recording; (vii) what to do in the case of difficulties or questions; (viii) what to do in the case of emergency. Participants were encouraged to ask questions, and – if they wished – to sample the recliner and/or stimuli prior to recording. These precautions, though unsophisticated, led to extremely low-noise recordings, yielding measurements with excellent reliability.

### 2.6 ABR wave I amplitude and growth

Following the methods of Mehraei et al. (2016), stimuli were monaural 80 μs broadband clicks, delivered at a rate of 9/s. The inter-stimulus interval was jittered by up to ±10 ms, in order to prevent the accumulation of stationary interference. Based on extensive piloting, click levels of 90, 96, and 102 dB peSPL were selected, with the aim of ensuring both participant comfort and clear wave I presence at all stimulus levels. The resulting three stimuli were interleaved on a click-by-click basis throughout the recording. Participants received 5200–5600 presentations at each level.

Bioelectrical activity between Cz and ipsilateral mastoid was filtered between 50 and 1500 Hz (fourth-order butterworth). Epochs were extracted (10 ms pre-stimulus to 10 ms post-stimulus) and rejected if activity exceeded the mean for the recording by more than two SDs. The remaining epochs were averaged and the resulting ABR corrected for any linear drift by subtracting a linear fit to the pre-stimulus baseline. Peak-to-trough wave I amplitudes were obtained using an automated peak-picking algorithm. The algorithm defined the peak of wave I as a local maximum occurring within a pre-specified time window. For stimuli delivered at 90 dB peSPL, the window was 1.7–2.5 ms after stimulus peak; at 96 dB peSPL, 1.6–2.3 ms; at 102 dB peSPL, 1.45–2.15 ms. If no maximum was found (as was true in one of 183 waveforms), wave I peak was taken to be the highest point of the waveform within the time window. The trough of wave I was defined as the lowest point of the waveform occurring 0.3–0.7 ms after the peak of wave I. Post-hoc subjective review verified that the algorithm had appropriately interpreted all waveforms, with the exception of one; for this waveform, the timing of the window was advanced, due to an unusually short-latency response.

Wave I growth was computed as in Mehraei et al. (2016), by fitting a straight line through the response amplitudes at the three stimulus levels, yielding a measure expressed in μV/dB. This would not control variability between individuals that is multiplicative (i.e., that scales amplitudes by a constant factor for each individual).

### 2.7 ABR wave V latency shift in noise

Following the methods of Mehraei et al. (2016), stimuli were monaural 80 μs broadband clicks embedded in broadband noise. Clicks were delivered at 93 dB peSPL and noise at 42, 54, and 66 dB SPL (as measured at the output of the transducer), with the aim of ensuring substantial shifts in latency for all participants without risking erasure of the response. Clicks were delivered at a rate of 9/s, jittered by up to ±10 ms. Presentation of the three noise levels was interleaved throughout the recording. Each block of noise had a duration of 2 s, including 10 ms onset and offset ramps. The offset ramp of each block was concurrent with the onset ramp of the next. Following the onset of each noise block, 80 ms elapsed before presentation of the first click. Participants received 3600–4000 presentations at each noise level.

Bioelectrical activity between Cz and ipsilateral mastoid was filtered between 50 and 1500 Hz (fourth-order butterworth). Epochs were extracted (10 ms pre-stimulus to 10 ms post-stimulus) and rejected if root-mean-square (RMS) activity exceeded the mean for the recording by more than two SDs. The remaining epochs were averaged and the resulting ABR corrected for any linear drift by subtracting a linear fit to the pre-stimulus baseline. Wave V latencies were obtained using an automated peak-picking algorithm. The algorithm defined the peak of wave V as the highest-amplitude local maximum occurring within a pre-specified time window. For responses obtained at a noise level of 42 dB SPL, the window was 5.7–6.9 ms after stimulus peak; at 54 dB SPL, 5.9–7.1 ms; at 66 dB SPL, 6.1–7.3 ms. Post-hoc subjective review verified that the algorithm had appropriately interpreted 97% of waveforms; for the remainder, picked peaks were manually corrected. ABR latency shift in noise was computed by fitting a straight line through the latencies of wave V at the three noise levels. Note that the three peak-picking windows overlapped substantially, and that no constraint was placed on the temporal order of the three peaks; nothing in the algorithm prevented a *decrease* in latency with noise level. This relatively unrestrictive approach was designed to ensure that the algorithm did not simply “force” plausible values of the latency-shift measure, thereby artificially inflating reliability. Despite this precaution, recorded values were predominantly positive (see section 3.2.3).

### 2.8 Envelope-following response

Stimuli were diotic 75 dB SPL transposed tones (Bernstein and Trahiotis, 2002) with carrier frequency 4000 Hz and modulation rate 105 Hz. In order to attenuate off-frequency contributions, as in Bharadwaj et al. (2015), tones were presented concurrently with a notched-noise masker (20–10000 Hz; notch width 800 Hz, centered on 4000 Hz), realized separately for each trial and applied at a signal-to-noise ratio (SNR) of 20 dB (broadband RMS). Stimuli had a duration of 400 ms, with the addition of 10 ms onset and offset ramps, and were presented at a rate of 1.25/s. Inter-stimulus interval was jittered by up to ±10 ms. Tones were of two modulation depths: 0 dB (full modulation) and −6 dB (shallow modulation). Each of these tones was presented 990 times, half in each polarity. The resulting four stimuli were interleaved throughout the recording, in the sequence 0 dB; −6 dB; 0 dB inverted; −6 dB inverted.

Bioelectrical activity between Cz and C7 was extracted for epochs extending from 8 to 408 ms after the end of the stimulus onset ramp. For each stimulus modulation depth and polarity, epochs were rejected if their RMS activity exceeded the 90^th^ percentile for the recording. The remaining epochs were averaged and the responses to opposing polarities summed, emphasizing the response to the temporal envelope of the stimulus. Each resulting EFR was subjected to a discrete Fourier transform to yield the response amplitude (at the 105 Hz modulation frequency) and an estimate of the noise floor (based on activity in 10 adjacent frequency bins: 87.5–97.5 Hz and 112.5–122.5 Hz).

Following Bharadwaj et al. (2015), we analyzed not only EFR amplitude but also a difference measure: the difference in EFR amplitude between the two stimulus modulation depths. This measure is closely related to the “EFR slope” reported by Bharadwaj and colleagues, though based on a two-point rather than a three-point function. Sensitivity to synaptopathy rests on the assumption that high-threshold fibers are preferentially damaged, and that these fibers are especially important for the encoding of stimuli with shallow modulations. Hence, ears with synaptopathy should exhibit unusually steep EFR growth with modulation depth.

### 2.9 Middle-ear-muscle reflex thresholds

MEMR thresholds were measured monaurally using a GSI Tympstar diagnostic middle-ear analyzer. The probe was encompassed in a Grason KR-Series clinical ear tip and delivered a probe tone at 226 Hz. Elicitors were ipsilateral pulsed pure tones at 1, 2, and 4 kHz with a duration of 1.5 s. Prior to measurement, the participant was instructed on the following: (i) basic setup and purpose of the test; (ii) sound quality of the elicitors; (iii) timing of the elicitors; (iv) timing of the response periods; (v) importance of artifact-free response periods; (vi) possible sources of measurement artifact (e.g. swallowing, movement, vocalization); (vii) test duration; (viii) importance of avoiding loudness discomfort; and (ix) verbal and non-verbal means of halting the test.

Reflex thresholds were determined by observing changes in middle-ear compliance following presentation of the elicitors. A reflex response was defined as a reduction in compliance of 0.02 ml or greater with appropriate morphology and no evidence of significant measurement artifact. If significant measurement artifact was observed during the response period, the presentation was repeated. For each threshold measurement, elicitor level commenced at 70 dB HL and ascended in 5 dB steps until a response was observed. Elicitor level then decreased by 10 dB and ascended in 2 dB steps until threshold was obtained. Threshold was defined as the lowest elicitor level at which three clear responses were observed over the course of three, four, or five artifact-free presentations. In addition to the thresholds at each frequency, we also analyzed a difference measure: the difference (in dB) between the thresholds at 1 and 4 kHz. It was reasoned that this measure might offer sensitivity to noise-induced damage, which tends to predominantly affect the 3–6 kHz region (Coles et al., 2000).

MEMR data are missing for one participant, due to poor fit of the probe tip in the ear canal. For another participant, an adequate fit was obtained at the first session but could not be achieved at the second; her MEMR data are omitted from the reliability analysis only.

### 2.10 Analysis

Test-retest reliability of each measure was assessed by computation of an ICC: the ratio of true variance to the sum of true variance and error variance. The specific form of ICC employed was a one-way, random-effects, single-rater ICC - denoted ICC(1,1) by Shrout and Fleiss (1979). Use of a one-way, random-effects model yields a more conservative estimate of reliability than other forms of ICC, and effectively regards each test session for each participant as a separate random judge sampled from the population, acknowledging that different test sessions involve differences in time of day, participant state, electrode placement, and so on. In Sections 3, 4, descriptors of reliability (“moderate”, “good”, etc.) follow the conventions of Koo and Li (2016).

After confirming normality of distribution, possible relations between the various proxy measures of synaptopathy were assessed by computation of Pearson's correlation coefficients (see Section 3.3 for comparisons).

## 3 Results

### 3.1 Measures of cochlear mechanical function

**Fig. 1:**
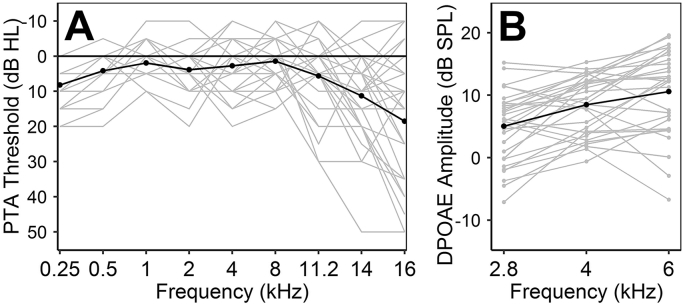
Measures of cochlear mechanical function for all participants. Mean values are given in black. A. Pure-tone audiometric thresholds. B. DPOAE amplitudes.

Fig. 1A illustrates pure-tone audiometric thresholds, which were ≤20 dB HL for all participants at frequencies from 0.25 to 8 kHz. Fig. 1B illustrates DPOAE amplitudes at 2.8, 4, and 6 kHz – frequencies that fall within the range typically associated with noise-induced hearing damage (Coles et al., 2000). At these frequencies, 97% of response SNRs (not shown in the figure) exceeded 6 dB.

### 3.2 Reliability of proxy measures of synaptopathy

#### 3.2.1 ABR wave I amplitude

**Fig. 2:**
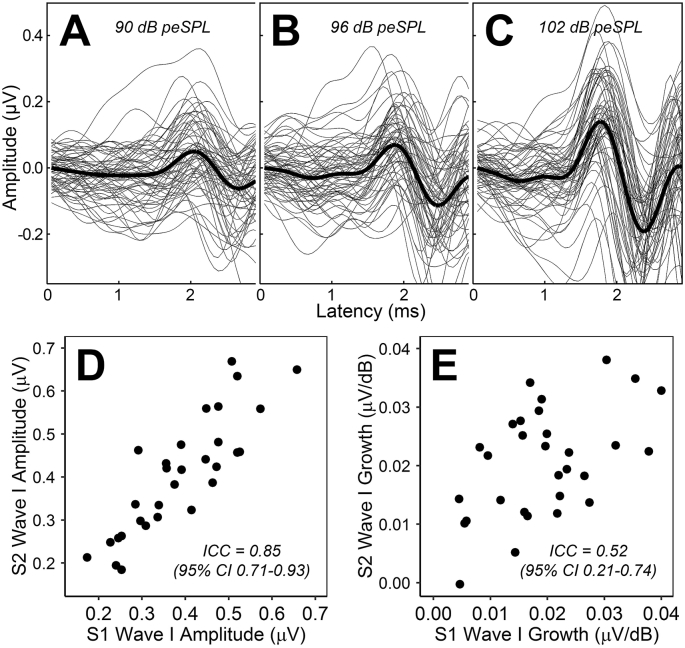
ABR wave I amplitude measures. A-C. Individual and grand average waveforms at click levels of 90, 96, and 102 dB peSPL. D. Comparison of wave I amplitude (at 102 dB peSPL) across the two test sessions. E. Comparison of wave I growth across the two test sessions.

Fig. 2C shows wave I of the ABR in response to 102 dB peSPL clicks for all participants. Mean wave I amplitude was 0.40 μV (range 0.17–0.67). Fig. 2D compares the amplitudes recorded at the first and second test sessions. Reliability was good, nearing excellent: ICC = 0.85 (95% CI = 0.71–0.93).

#### 3.2.2 ABR wave I growth

The change in wave I amplitude with increasing stimulus level was always positive, with exception of one participant, who presented near-zero growth at both test sessions. The growth of amplitude with stimulus level is also apparent in the grand average waveforms (Fig. 2, upper panels). Mean growth was 0.02 μV/dB (range = −0.0002 to 0.04). Reliability of the measure (Fig. 2E) was moderate, nearing poor: ICC = 0.52 (95% CI = 0.21–0.74).

#### 3.2.3 ABR wave V latency shift in noise

**Fig. 3:**
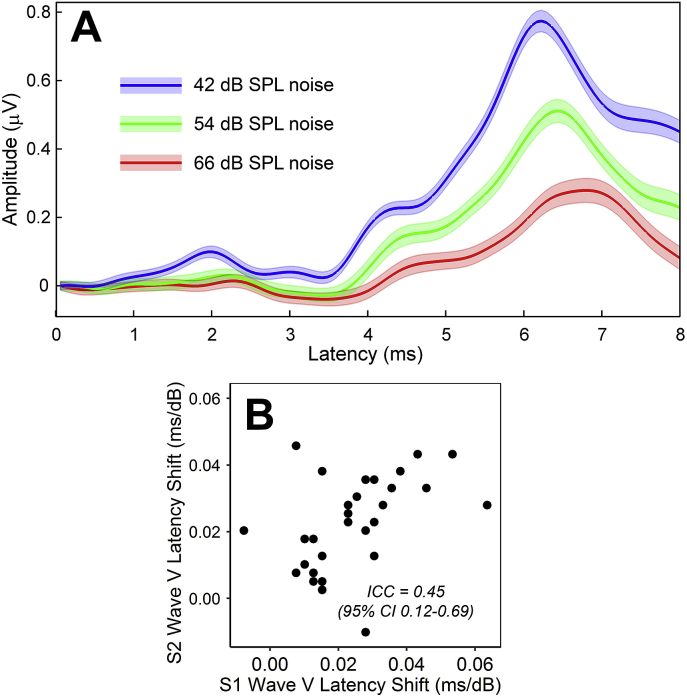
ABR latency shift in noise. **A.** Grand average waveforms. Shaded areas represent the standard error of the man. **B.** Comparison of the wave-V-latency-shift measure across the two test sessions.

Fig. 3A presents grand average ABRs at the three noise levels. In 97% of measurements, response latency increased with noise level, as expected. Mean latency shift was 0.024 ms/dB (range −0.01 to 0.064). Reliability of the measure (Fig. 3B) was poor: ICC = 0.45 (95% CI = 0.12–0.69). In contrast, reliability of raw ABR wave V latency in 42 dB SPL noise was good, nearing excellent: ICC = 0.86 (95% CI = 0.73–0.93).

#### 3.2.4 EFR amplitude

**Fig. 4:**
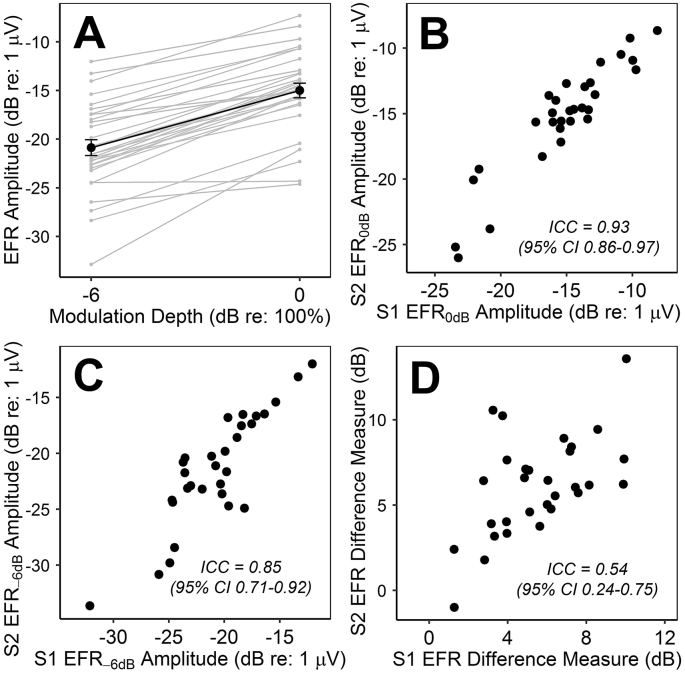
EFR measures. **A.** Response amplitudes for all participants. Black points and error bars represent the mean ± standard error of the mean. **B-D.** Comparison across test sessions of (B) response amplitudes at the full stimulus modulation depth, (C) response amplitudes at the shallow modulation depth, and (D) growth of response amplitude with increasing modulation depth.

Response SNR exceeded 6 dB for 100% of responses to the fully modulated stimulus and for 87% of responses to the shallow stimulus modulation depth. Mean EFR amplitudes (Fig. 4A) at the full and shallow depths were −15 and −20.9 dB re: 1 μV, respectively. Reliability at the full modulation depth (Fig. 4B) was excellent: ICC = 0.93 (95% CI = 0.86–0.97). Reliability at the shallow depth (Fig. 4C) was good, nearing excellent: ICC = 0.85 (95% CI = 0.71–0.92).

#### 3.2.5 EFR difference measure

The change in EFR amplitude with increasing modulation depth was always positive, with exception of one participant, who presented near-zero values at both test sessions, despite response SNRs >6 dB. Mean difference in response amplitude between the modulation depths was 5.87 dB. Reliability of this EFR difference measure (Fig. 4D) was moderate: ICC = 0.54 (95% CI = 0.24–0.75).

#### 3.2.6 MEMR thresholds

Mean thresholds measured at 1, 2, and 4 kHz were 85.3, 86.6, and 90.0 dB HL, respectively. For six participants, thresholds at 4 kHz exceeded the maximum output of the middle-ear analyzer (100 dB HL) at one (n = 3) or both (n = 3) test sessions. These participants were omitted from the 4 kHz reliability analysis; for the purposes of the correlational analysis, the affected thresholds were ascribed a value of 102 dB HL.

**Fig. 5:**
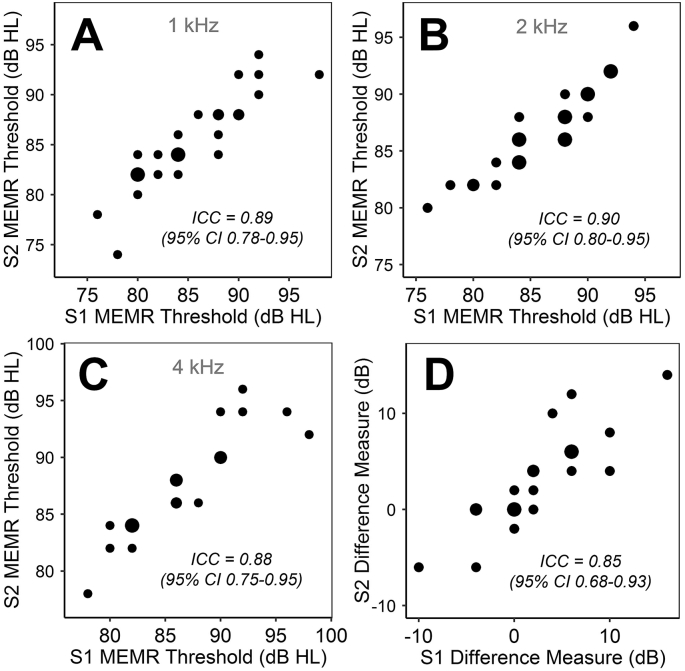
Comparison across test sessions of the four MEMR measures. Point size represents number of observations. **A.** 1 kHz thresholds. **B.** 2 kHz thresholds. **C.** 4 kHz thresholds. **D.** Across-frequency threshold difference (4 kHz–1 kHz).

Reliability of the 1 kHz thresholds (Fig. 5A) was good, nearing excellent: ICC = 0.89 (95% CI = 0.78–0.95). Reliability of the 2 kHz thresholds (Fig. 5B) was excellent: ICC = 0.90 (95% CI = 0.80–0.95). Reliability of the 4 kHz thresholds (Fig. 5C) was good, nearing excellent: ICC = 0.88 (95% CI = 0.75–0.95). Values of the MEMR difference measure (4 kHz threshold – 1 kHz threshold) ranged from −10 to 18 dB; reliability was good, nearing excellent (Fig. 5D): ICC = 0.85 (95% CI = 0.68–0.93).

### 3.3 Relations between proxy measures of synaptopathy

**Fig. 6:**
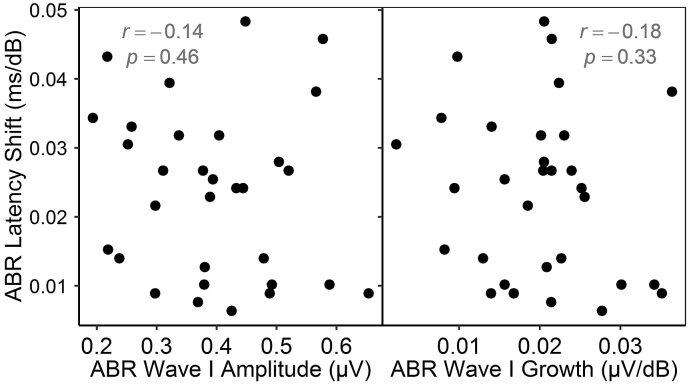
ABR latency shift in noise *vs* ABR wave I amplitude and growth. No significant correlations are evident.

**Fig. 7:**
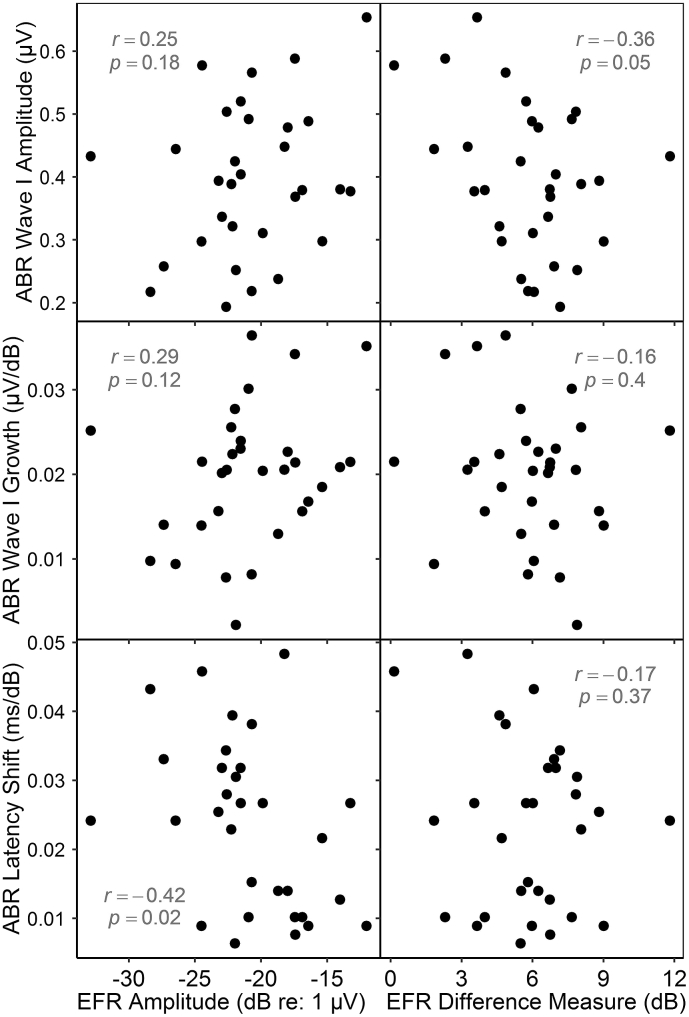
ABR *vs* EFR measures. Plotted EFR amplitudes (left column) are those obtained at the shallow stimulus modulation depth. A marginally significant correlation (uncorrected) is evident between ABR wave I amplitude and the growth of EFR amplitude with modulation depth, but would not survive correction for multiple comparisons. An apparent correlation between EFR amplitude and ABR wave V latency shift runs counter to the predicted direction.

**Fig. 8:**
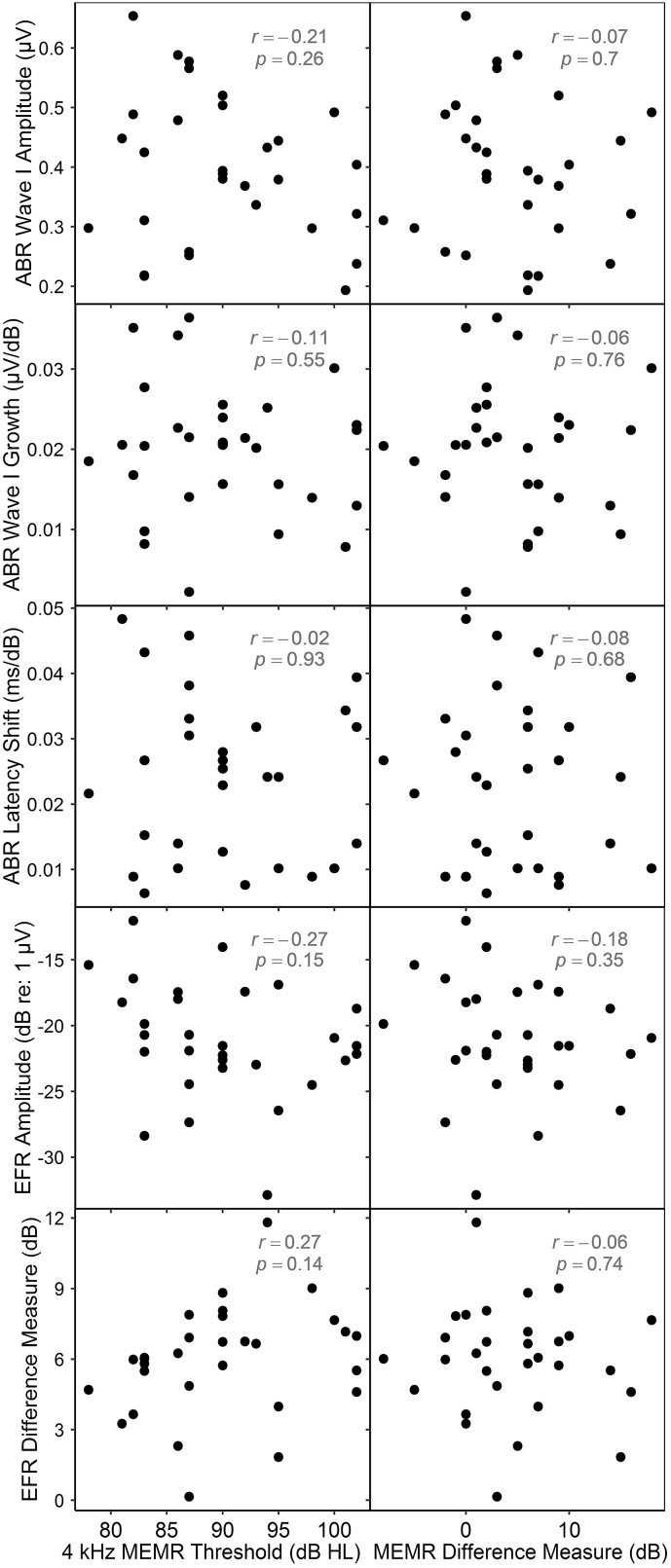
Electrophysiological measures *vs* MEMR threshold measures. Plotted EFR amplitudes (fourth row) are those obtained at the shallow stimulus modulation depth. No significant correlations are evident.

Eighteen comparisons were conducted, illustrated in Fig. 6, Fig. 7, Fig. 8.

#### 3.3.1 ABR amplitude measures vs ABR wave V latency shift in noise

No significant relation was evident between ABR wave V latency shift in noise and either ABR wave I amplitude or ABR wave I growth (Fig. 6).

#### 3.3.2 ABR measures vs EFR measures

Six comparisons were conducted, pairing each of the two EFR measures with each of the three ABR measures (see Fig. 7). Four yielded no significant relation. Comparison of ABR wave I amplitude with the EFR difference measure yielded a marginally significant correlation (*r* = −0.36, *p* = 0.05, uncorrected), but this relation was clearly too weak to survive correction for multiple comparisons. A stronger correlation was evident between EFR amplitude and ABR latency shift in noise; however, this relation ran in the opposite-from-predicted direction, and would not withstand correction in any case.

#### 3.3.3 ABR measures vs MEMR measures

MEMR thresholds at 4 kHz and the MEMR difference measure were each compared with the three ABR measures (Fig. 8, upper six panels). No significant correlations were evident.

#### 3.3.4 EFR measures vs MEMR measures

The two MEMR measures were compared with the two EFR measures (Fig. 8, lower four panels). No significant correlations were evident.

## 4 Discussion

### 4.1 Highly reliable measures of ABR and EFR amplitude are possible in humans

The reliability of ABR wave I amplitude is not widely documented in the literature; past research has tended to focus on latency measures. Bidelman et al. (2018) assessed the reliability of ABR amplitude, but only for wave V, which was found to be moderately reliable (ICC = 0.65). However, a previous study by our lab examined the test-retest reliability of ABR wave I amplitude using clinical recording equipment and yielded an ICC of 0.85 (Prendergast et al., 2018). The present study differed in methodology (e.g. use of research-grade recording equipment and a lower click level), yet produced a near-identical ICC. Our findings suggest that the amplitude of ABR wave I can be a highly reliable measure, if care is taken to ensure consistency in electrode placement, participant state, and other factors influenced by the investigator. Since lower reliability has been reported elsewhere, researchers adopting ABR amplitude measures would be well advised to undertake preliminary assessments of the reliability of their own ABR measurements.

Results for EFR amplitude were even more favourable: good-to-excellent reliability, depending on stimulus modulation depth. These values are in line with those reported by Bidelman et al. (2018) for frequency-following-response amplitude. It is worth noting that our EFRs were obtained using a simple, single-channel recording setup, suggesting that multi-channel recordings are not necessary to ensure response reliability.

### 4.2 Differential ABR and EFR measures exhibit poor reliability

The reliability of the ABR and EFR difference measures was poor-to-moderate, despite good-to-excellent reliability of the raw ABR and EFR measures. Of course, reliability depends on not only the magnitude of measurement error, but also the heterogeneity of the true values in the study population. If a difference measure is effective in eliminating non-synaptopathic sources of variability and is applied in a homogeneous population, varying little in presence of synaptopathy, then both between-subject variance and reliability are bound to be low. Hence, it is reasonable to ask whether the low reliability observed in the present study is predominantly the result of low true variance, rather than high measurement error. This appears to be true in the case of the EFR difference measure, whose between-subject SD (2.9 dB) is far lower than that of raw EFR amplitude (5.1 dB), despite similar within-subject SDs (∼1.8 dB). The same is not true of the ABR growth measure; when expressed as the amplitude difference (in μV) corresponding to a 12 dB increase in stimulus level, this measure's between-subject SD is slightly lower than that of raw wave I amplitude (0.11 c.f. 0.13 μV), but its within-subject SD is markedly higher (0.78 c.f. 0.49 μV).

However, regardless of its sources, the low reliability of all three difference measures casts doubt on their value, at least when used in samples similar to our own. Previous studies have employed these difference measures in small (n ≈ 30) samples of normally hearing young adults, and reported possible evidence for synaptopathy (Bharadwaj et al., 2015; Mehraei et al., 2016). Yet the consistently low ICCs reported here suggest that all three difference measures lack the capacity to distinguish powerfully between members of such populations. The EFR measure might be more valuable in populations with greater true variance – perhaps older adults, and/or those with a greater range of noise exposures – and all would have greater utility in much larger samples. Nevertheless, we urge caution to investigators considering the use of these difference measures in young humans with normal audiograms, and to those interpreting the results of such measures.

### 4.3 Highly reliable MEMR threshold measures are possible in humans

MEMR thresholds, measured using a clinical middle-ear analyzer, exhibited excellent or near-excellent reliability. Previous research in adult humans has indicated somewhat poorer reliability, especially for tests conducted on different days (Chermak et al., 1983). These results suggest that careful measurement techniques may be important in ensuring reliable responses (see Section 2.9).

The present study elicited MEMRs using pure tones, based on the reasoning that synaptopathy might be most prevalent in the 3–6 kHz region, where early noise damage is often manifest (Coles et al., 2000), and that use of a broadband elicitor might dilute its effects. This reasoning is supported by MEMR findings in a mouse model (Valero et al., 2018), in which sensitivity to synaptopathy was far greater with a narrowband than a broadband elicitor. It is worth noting that our choice of *probe* (226 Hz tonal) differed from the wideband probe used by Valero and colleagues; however, when these two approaches have been compared in adult humans, they have been shown to yield similar 4 kHz thresholds (Feeney et al., 2004; Keefe et al., 2010).

We also assessed the reliability of a difference measure, comparing thresholds at 1 and 4 kHz. The mouse data of Valero et al. (2018) suggest that such a measure might have value: synapse loss was highly correlated with MEMR threshold when elicitor frequency lay in a synaptopathic region (r = 0.89) and uncorrelated in a lower frequency region (r = 0.17). In the present data, the difference measure exhibited good reliability.

### 4.4 Purported proxy measures of cochlear synaptopathy do not correlate

We observed no correlations of interest between any of our proxy measures of cochlear synaptopathy. This was despite high reliability of the raw threshold and amplitude measures, wide between-subject variability in these measures, and use of a sample resembling those of previous studies of synaptopathy in humans. Of the 18 comparisons, two yielded apparent correlations: one that trended in the predicted direction but was marginal, and one that opposed the predicted relation; neither would withstand correction for multiple comparisons. Hence, we find no evidence that any of the measures assess the same underlying physiological processes. Of course, our sample size was small, and it is possible that correlations might have been evident with a greater number of participants. However, it is notable that even consistent trends are not evident in the data.

The lack of correlation between ABR latency shift in noise and ABR wave I growth contrasts with the findings of Mehraei et al. (2016), who reported a significant correlation (*r*^2^ = 0.44, *p* = 0.036, uncorrected). The two studies differ principally in sample size: n = 31 in the present study, n = 10 in the previous study. The present data for ABR latency shift in noise were also obtained using a higher click level (93 c.f. 80 dB peSPL), though it is unlikely that this factor could reduce sensitivity to synaptopathy. The studies used similar noise levels: Mehraei and colleagues presented noise at 42, 52, 62, 72, and 82 dB SPL and analyzed whichever conditions produced clear responses, most often 42–62 dB SPL. Finally, it is worth noting that wave I growth could only be measured in a subset of the participants enrolled by Mehraei and colleagues, and the resulting growth values are an order of magnitude lower than those of the present study.

Two broad explanations exist for the absence of correlations observed in our data: (a) most young women with normal audiograms lack variance in synaptopathy, and (b) most or all of the measures investigated in this study are insensitive to synaptopathy in humans. Possibility (a) should not be discounted, since straightforward translation of synaptopathic findings from animal models to humans is unlikely (Hickox et al., 2017). Human ears may possess superior resistance to noise-induced synaptopathy, given known interspecies differences (Dobie and Humes, 2017). Consequently, synaptopathy in humans might require relatively extreme exposures, or – as we have argued previously – might arise primarily in older adults, rather than the very young (Guest et al., 2017). Moreover, some degree of hair-cell loss is often present in animal models of synaptopathy (Hickox et al., 2017); since real-world noise exposures differ greatly from carefully titrated laboratory exposures, synaptopathy in young humans *without audiometric loss* may be rare. Despite these clear translational issues, several publications have reported possible evidence for synaptopathy in cohorts similar to that of the present study (Bharadwaj et al., 2015; Stamper and Johnson, 2015; Mehraei et al., 2016), though null results have been more commonplace (e.g. Fulbright et al., 2017; Grose et al., 2017; Prendergast et al., 2017). Hence, it is important to consider possibility (b), namely that most of the measures used in this study do not index synaptopathy. A logical question, then, is which of the procedures constitute *plausible* measures of synaptopathy.

The amplitude and growth of ABR wave I have been shown to relate to synaptopathy in numerous animal models. In humans, wave I amplitude is influenced by many sources of variability besides AN function, but can at least be measured reliably. Growth of wave I theoretically offers a means of managing non-synaptopathic variability, thereby offering greater sensitivity to loss of high-threshold fibers. However, our data indicate that this measure exhibits substantially poorer reliability, underpinned largely by measurement error.

ABR latency shift in noise is similarly unreliable and less well validated, but has at least been shown to relate to synaptopathy at the group level (Mehraei et al., 2016). However, it is not clear that, in humans, the shift in wave V latency has its basis in the auditory periphery. Increasing noise levels lead not only to longer wave V latencies but also to longer wave I—V interpeak intervals (Burkard and Hecox, 1987; Gott and Hughes, 1989; Burkard and Sims, 2002). Moreover, Burkard and Hecox saw greater increases in the wave III-V interval than the wave I-III interval, casting further doubt on Mehraei and colleagues’ peripheral interpretation. Confidence in wave V latency shift as a measure of synaptopathy would be strengthened by improved understanding of its potential bases in the central auditory system.

Interpretation of EFR measures of synaptopathy is similarly complicated by the issue of central influences. Since the EFR is a sustained response, it combines contributions from a variety of generators (Krishnan, 2006). In animal models, high modulation rates (∼1 kHz) are required to emphasize AN contributions and provide sensitive detection of synaptopathy (Shaheen et al., 2015; Parthasarathy and Kujawa, 2018). Human EFR studies of synaptopathy have used far lower modulation rates (Bharadwaj et al., 2015; Grose et al., 2017; Guest et al., 2018), yielding responses that are likely dominated by higher centers.

MEMR thresholds can be highly reliable in humans and have been shown to correlate closely with synaptic survival in mice (Valero et al., 2018). A shortcoming of these measurements in humans is that between-subject variability – likely due to factors besides synaptopathy – is extremely high: in the present cohort, 1 kHz thresholds spanned a 24 dB range. Our across-frequency difference measure, which was designed to manage this variability, is highly reliable. However, validity of the difference measure rests on the assumption that thresholds at 1 and 4 kHz are not differentially affected by factors other than synaptopathy, and this is by no means certain. The high thresholds observed in some participants at 4 kHz might reflect synaptopathy; alternatively, they might reflect other factors that vary across frequency, such as reflex adaptation (Gelfand, 2009) or differences in pre-neural function.

## 5 Conclusion

The number of proxy measures of cochlear synaptopathy employed in humans has exploded in recent years, extending into double figures. Here, we investigated the reliability and interrelations of several of these measures, in a cohort of young, audiometrically normal women. Results indicate that raw ABR and EFR amplitudes can be highly reliable, given careful measurement techniques, as can MEMR thresholds. In contrast, all differential ABR and EFR measures exhibited poor or near-to-poor reliability. No significant correlations were evident between measures, despite their application in a sample resembling those of previous studies reporting possible evidence for synaptopathy. These findings suggest that many proxy measures of cochlear synaptopathy should be regarded with caution, at least when employed in young adults with normal audiograms.

## Appendix A Supplementary data

Data Profile Data Profile
